# Giant desmoid tumour mimicking recurrent uterine myoma in a nulliparous young Nigerian: a case report

**DOI:** 10.1186/s13256-022-03558-6

**Published:** 2022-08-27

**Authors:** Obinna Chukwunwike Njoku, Chisara Cyprian Umezurike

**Affiliations:** 1 Royal Tropical Institute, Amsterdam, Netherlands; 2 Present Address: Kahabiri Specialist Hospital, Aba, Abia State Nigeria; 3 Nigerian Christian Hospital, Aba, Nigeria

**Keywords:** Desmoid tumor, Myoma, Nullipara

## Abstract

**Background:**

Desmoid tumors are rare lesions. Although they demonstrate tumor characteristics, such as infiltrative growth and tendency towards local recurrence, they lack the ability to metastasize. To date, the cause of desmoid tumors is unknown. They can occur in both sexes, but predominant slightly in women, including nulliparous women, of reproductive age, but mostly during and after pregnancy.

**Case presentation:**

A 36-year-old nulliparous Nigerian woman presented with a large desmoid tumor of the anterior abdominal wall, mimicking recurrent leiomyoma. At presentation, she had a painless abdominal mass for 1 year, which was first noticeable as a small induration that progressively increased in size. The patient had a previous surgical history of open myomectomy for symptomatic fibroids of 3 years duration, prior to presentation. Treatment comprised a complete excision of the tumor with a wide margin and partial omentectomy and the anterior abdominal wall closed in layers, though without prosthesis. The patient subsequently developed incisional hernia.

**Conclusions:**

Large desmoid tumors may be misdiagnosed or mistaken for uterine leiomyoma or other abdominal or pelvic tumors. Attention should therefore be paid to detailed patient history and systematic clinical evaluation. To guard against incisional hernia associated with surgical resection of huge desmoid tumors, mesh reconstruction is recommended.

## Background

Desmoid tumors originate from mesenchymal tissues as a result of disproportionate fibroblast proliferation [1]. They are relatively uncommon lesions, representing up to 0.03% of all neoplasms and 3% of all soft tissue tumors [2], with an incidence of five to six cases per 1 million general population per annum [3] at the peak age of 30–40 years [4]. They occur slightly more often in females than in males [5]. Desmoid tumors have the potential to grow, become aggressive and infiltrate the immediate surrounding tissues and organs. Therefore, they are also referred to as aggressive fibromatosis. They can develop virtually in any part of the body but are usually found in the limbs, abdomen, and thorax [6, 7]. However, according to World Health Organization, despite demonstrating the characteristics of infiltrative growth and tendency towards local recurrence, desmoid tumours lack the ability to metastasize [8].

The cause of these tumors is unknown, but studies have shown that desmoid tumours most likely occur in individuals with a family history of colon cancer and polyposis coli [7]. In postpartum women, the tumors frequently develop from the rectus abdominis muscle as well as from incisional abdominal scars [9, 10]. Its occasional relatively aggressive nature and propensity for recurrence account for the challenges associated with the treatment of this rare tumor. Patient age and location of tumor determine the treatment options, which range from chemotherapy to surgery to radiation [7]. However, a desmoid tumor frequently presents as a painless enlarging mass, occasionally with ulceration on late presentation. Notwithstanding, complete resection with tumor-free margin is a typical treatment option [11] Here, we report the case of a 36-year old Nigerian woman with giant desmoid tumour mimicking recurrent uterine leiomyoma, who subsequently developed incisional hernia.

## Case presentation

A 36-year-old nulliparous Nigerian petty trader presented at the gynecology outpatient clinic with a painless abdominal mass of 1-year duration. The mass first appeared as a small induration and progressively increased in size. She had menarche at age 14 years, with 3 days of menstruation in a 28-day cycle. There was no history of dysmenorrhea, menorrhagia and bowel or urinary change, and no significant past medical history although she was taking the antihypertensive amlodipine (tablet, 10 mg daily) for hypertension. She had a surgical history of open myomectomy for symptomatic fibroids 3 years prior to presentation. There was no personal or family history of diabetes mellitus, asthma, cancer or sickle cell disease. She defined herself as a social drinker and non-smoker (no history of smoking). The results of a prior ultrasound examination at a different hospital suggested adenomyosis, and based on this scan, she was offered a hysterectomy at that hospital prior to her presentation at our own facility. General and neurological examinations were unremarkable, with a pulse rate of 74 beats per minute, a blood pressure of 130/80 mmHg, and a body temperature of 36.2 °C. Abdominal with bimanual pelvic examination revealed a giant abdominopelvic mass, equivalent to a 36-week gestational uterus, extending in all directions to fill the abdominal cavity. It was not attached to the overlying structures but seemed to have originated from the pelvic cavity.

**Fig. 1:**
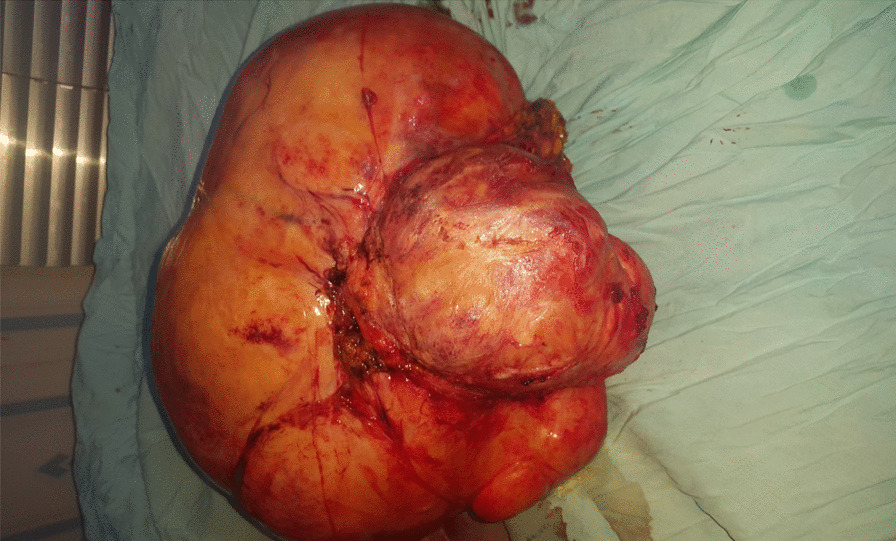
Excised huge desmoid tumor, weighing 6.5 kg, with a diameter of 40 cm

**Fig. 2:**
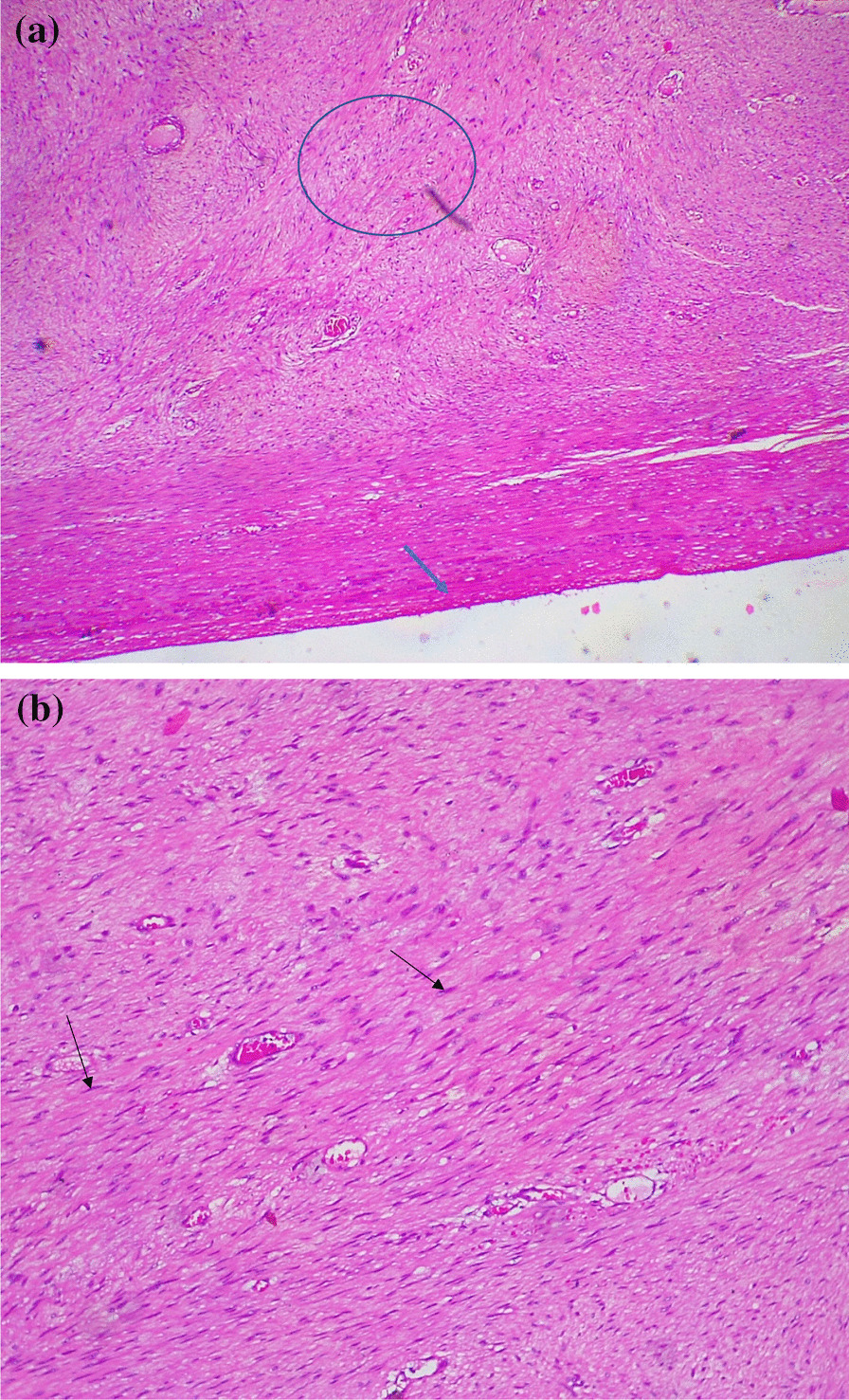
Histology study. **a** Low-power view shows a mesenchymal tumor with pseudo capsule (thick blue arrow). The cells are spindle to stellate and arranged in sweeping fascicles and vague storiform pattern (thin blue circle). Staining: hematoxylin and eosin (H/E); magnification: ×4. **b** Cells are spindle to stellate in shape (thin black arrow) with no pleomorphism or mitotic activity. Staining: H/E; magnification ×10

Subsequently, based on evidence of this abdominopelvic mass, we carried out a preoperative work-up, with the results showing a packed cell volume (PCV) of 36%, fasting blood sugar of 80 mg/dl, blood group 0^+^ (positive), sero-negativity for hepatitis B and C, and normal values for urinalysis, electrolytes, urea, and creatinine. The abdominopelvic scan revealed an anteverted, empty, and markedly bulky uterus that measured about 283 × 156 mm, with the fundus close to the xiphisternum. The endometrial stripe measured about 4 mm in thickness, with what appeared to be diffuse enlargement of the uterine myometrium with no succinct myometrial mass. There was no adnexal mass and no fluid in the Pouch of Douglas. The visceral organs showed noting of significance. The diagnosis was adenomyosis uteri. A computed tomography scan and magnetic resonance imaging were not performed due to the unavailability of these facilities. Two units of whole blood were cross matched for her, and she then underwent an exploratory laparotomy for the excision of the tumor. Intra-operation findings included a huge 6.5-kg abdominal mass of firm consistency and 40 cm in diameter (Fig. 1). There was adhesion of the omentum with markedly enlarged omental vessels. The uterus, fallopian tubes, and ovaries appeared to be normal. There was no retroperitoneal involvement. However, there was an extension of the tumor to the left sub-umbilical part of the abdominal wall. Complete excision of the tumor with a wide margin and partial omentectomy was carried out, and the anterior abdominal wall was closed in layers without prosthesis. The postoperative period was uneventful, and she was discharged on the seventh postoperative day. The histology study showed intra-abdominal desmoid-type fibromatosis with an unremarkable fibrofatty tissue stalk excision margin (Fig. 2a, b). Ten months later, during follow-up visit, the patient was found to have developed incisional hernia, which was also repaired.

## Discussion and conclusions

We present here a case of massive desmoid tumor in a nulliparous woman with a previous history of open myomectomy. It was a clear-cut case of diagnostic masquerader that mimicked a recurrent uterine leiomyoma and indeed any other abdomino–pelvic tumors. Hence, this diagnosis was a retrospective one.

Desmoid tumors are deep benign fibromatoses that can originate from the fascia and aponeurosis of the muscle with local infiltrating growth. They can occur in any part of the body [12] and can be either slow-growing or aggressive forms. The slow-growing tumors are not associated with familial adenomatous polyposis (FAP) and are typically sporadic, while the aggressive ones are typically associated with FAP [13]. The slow-growing tumors commonly occur in the shoulder girdle or the abdominal wall, with the latter more common in young females during pregnancy or within the first year after childbirth [14, 15], as in our patient, although she is nullipara. However, young nulliparous women using contraceptives with a history of colectomy are at higher risk of developing a desmoid tumor [16].

Clinicians, especially those working in remote areas, are often clinically challenged when presented with abdominal tumors. Misdiagnosis or confusion in providing a reliable diagnosis may be associated with hasty evaluation, the large size of the tumor, uncommon site, and conflicting outcomes from different analytical and diagnostic tools, such as computed tomography and ultrasound scan, among others. In female patients, a large number of the pelvic masses regularly encountered by clinicians are of various tumour types, such as leiomyoma, desmoid tumors, ovarian cyst, and ovarian malignant tumour, leading to possible misdiagnosis [17]. Our patient had the experience of being offered hysterectomy at a previous institution following the ultrasound scan that suggested adenomyosis. Uncommon pelvic tumours may also be encountered, such as desmoid tumors, mesothelioma, among others. Desmoid tumors are able to manifest as huge pelvic mass, with the clinical symptoms concealed due to the slow growth of the tumor. Desmoid tumors that mimic intra-abdominal tumors have been misdiagnosed, as reported by clinicians [17]. Therefore, a histopathology evaluation should be given priority for a definitive diagnosis [11]. Our case was a diagnostic dilemma until the histology report was provided.

The pathobiology of desmoid tumors is poorly understood, and as the behavior of these tumours is erratic, there is no standardized surgical or medical therapy [18]. Therefore, in 2005, Church et al, provided a significant approach for the management of such condition [19]. A clinical staging system was proposed based on tumor growth, size, and existence of symptoms and complications. Stage I, II and III are amenable to various forms of therapy, such as non-steroidal anti-inflammatory drugs (NSAIDs), selective tyrosine kinase inhibitor, anti-estrogens, chemotherapy, and surgical resection [18], as was applicable to own patient. Thus, she underwent surgical resection. Stage IV tumors, which are > 20 cm, fast growing, and with several symptoms, are associated with increased disease and death rates. Moreover, medical treatment therapy is not feasible as much time is needed to achieve the expected benefits, while surgical resection may be impossible due to the encasement of vital organs [18].

In addition to the association between desmoid tumors and trauma, estrogen therapy, FAP and Gardner syndrome, there is a relationship between the tumor and patient’s previous history of abdominal or pelvic surgery [11]. This was applicable to our patient who had a past surgical history of open myomectomy for symptomatic fibroids. It is also pertinent to take note of rectus abdominis lesions in the differential diagnosis, such as lymphoma, fibrosarcoma, hematoma, leiomyosarcoma, among others [11].

Incisional hernia and abdominal wall bulging, among others, are complications associated with the surgical resection of abdominal desmoid tumors. These are delayed complications, noted during the post-operative patient follow-up visit [20]. Experts suggest that repair of abdominal wall defects could be adequately accomplished with prosthetic mesh reconstruction, with resultant excellent functional results [1]. This expert advice was not implemented in the management of own case, with the consequence of incisional hernia. However, studies have shown that due to infections and other complications associated with synthetic or biological mesh, autographs are also recommended. Autographs are said to be associated with fewer, less severe complications, such as graft degradation and or infection and local or systemic rejection reactions [21].

Large-sized desmoid tumours may be misdiagnosed or mistaken for recurrent leiomyoma or other abdominal or pelvic tumors. Such misdiagnoses are often associated with a hasty and poor evaluation, tumor size, and uncommon tumor site. Therefore, a detailed history, systematic physical examination, and good knowledge of the differential diagnosis of desmoid tumours are key to overcoming this challenge. To guard against incisional hernia associated with surgical resection of huge desmoid tumours, mesh reconstruction is recommended.

## Data Availability

Not applicable.
